# Fine scale spatial variability in the influence of environmental cycles on the occurrence of dolphins at coastal sites

**DOI:** 10.1038/s41598-019-38900-4

**Published:** 2019-02-22

**Authors:** Oihane Fernandez-Betelu, Isla M. Graham, Thomas Cornulier, Paul M. Thompson

**Affiliations:** 1 School of Biological Sciences, Lighthouse Field Station, University of Aberdeen, Cromarty, United Kingdom; 2 School of Biological Sciences, University of Aberdeen, Aberdeen, United Kingdom

## Abstract

Environmental cycles often influence the presence of animals, creating patterns at different temporal scales, which may mean that their effects overlap and/or interact. Interactions between diel and seasonal cycles have been reported to influence fish behaviour but little is known about such interactions in marine top predators. Here, we studied the combined effect of seasonal, tidal and diel cycles on the occurrence of bottlenose dolphins (*Tursiops truncatus*) within a Marine Protected Area in Scotland. Our analyses were based on echolocation detections from passive acoustic devices (CPODs) deployed at three coastal sites between 2010 and 2016. We described patterns of dolphins’ occurrence using circular statistics and then used generalised additive mixed models to explore the relative importance of each cycle and any interactions between them. We found site-specific cyclical patterns of presence that remained constant across years. There was a highly significant interaction between seasonal and diel cycles at two sites around deep channels, where occurrence was diurnal in summer but became nocturnal in autumn. The study demonstrates the highly plastic behaviour of bottlenose dolphins and shows a previously unreported behaviour that has management implications for this and other marine protected areas.

## Introduction

Environmental cycles create rhythmic patterns that modify the abiotic conditions of ecosystems, and biological rhythms that match these cycles are widespread^1^. In mid and high latitudes, seasonal changes in temperature and day length lead to major changes in the abundance and distribution of many species across trophic levels^2–4^. The daily light-dark cycle results in diel activity patterns which may be diurnal, nocturnal or crepuscular. The trade-off between foraging success and predation risk is considered the most critical influence on diel behaviour^5,6^. In coastal environments, the tidal cycle also causes dramatic physiochemical changes that result in periodic movements of many species. The main driver of tidal migrations in coastal areas is the avoidance of unsuitable conditions, but tidal currents are also used for transport, feeding, predator avoidance and reproduction^7^.

Since these environmental cycles can create patterns at different temporal scales, their effects may interact. The interaction most often studied in marine organisms is the one between seasonal and diel cycles. In winter, animals must choose between adapting to local, often harsh, conditions and migrating to more favourable habitats. For animals with a flexible diel behaviour, one strategy they can follow to adapt to local conditions is to modify their diel activity patterns. Examples of such seasonal changes in diel activity patterns can be found across many taxa: see Hut *et al*. for a review^8^. The diel behaviour of fish is known to be very plastic^9^. For example, salmonids and sea bass may be diurnal in summer but nocturnal in winter^10^, while other species such as cod exhibit more complicated seasonal diel shifts^11^.

The influence of environmental cycles on top predators is not only direct, they are also affected by the rhythmicity of their prey. Therefore, their cyclic patterns of behaviour often match those of their prey^12–14^. In the marine environment, predators have developed highly flexible behaviour in response to the dynamic environment in which they live^15^. As an example of that flexibility, marine mammals exhibit a full spectrum of responses to environmental cycles. Studies of the effect of seasonal, tidal and diel cycles on their behaviour have produced highly variable results^16,17^. However, whilst interactions between different environmental cycles have been described for some of their prey, there is a lack of information about more complex responses in marine mammals.

Understanding behavioural variation due to natural cycles is not only key to our knowledge of animal behaviour in the wild but, for protected species, it is also key to their conservation management. In recent years, many Marine Protected Areas (MPA) have been designated to protect marine mammals^18^. More dynamic approaches with flexible spatial and temporal boundaries that protect the core areas have been recommended for mobile species^19^. Habitat modelling has been used as a method to identify key areas for target species^20^ and many authors have highlighted the importance of including dynamic environmental variables because static physical features on their own do not capture the complexity of the habitat selection process^17,21^. For instance, without including temporal variables in the models, it was found that a sandy bank was an important site for harbour porpoises in the Moray Firth^22^. However, once the diel cycle was included, it was found that adjacent muddy areas were also important habitats for them during the night^23^. The management of potential stressors relies on accurate information about the distribution of focal species; consequently, the inclusion of both spatial and temporal variables becomes necessary to implement efficient protection measures for highly mobile species.

The present study aims to explore the combined effects of seasonal, tidal and diel cycles on the presence of bottlenose dolphins in an MPA in the Moray Firth, NE Scotland. Previous studies in this area have identified relationships with some of these cyclic variables, and there is evidence that there may be fine-scale spatial variation in these patterns^24,25^ that could have implications for managing this protected population. Early boat-based visual surveys demonstrated that individuals from this population remained in the area all year round, with a higher number of encounters during summer^26^. However, whilst subsequent studies in one tidal channel detected relationships with both time of day and tidal cycles^25,27^, no tidal or diurnal effects were identified at a second nearby channel with similar physical characteristics^24^. Determining whether this represents real spatial variation in the importance of these different environmental variables was not possible because these studies were undertaken at different times, using slightly different methodologies. Furthermore, these earlier assessments of diel patterns were constrained to analyses of daylight observations, preventing an analysis of the full diel cycle and the interactions between these different cycles.

Long-term passive acoustic studies provide unique opportunities for exploring variation in the occurrence and behaviour of cetaceans across whole seasonal and diel cycles that would be impossible to sample representatively using visual methods^28,29^. Here we use a 6 year time series of passive acoustic recordings to compare temporal patterns of dolphin occurrence at three sites within the Moray Firth, that include the two potentially contrasting sites studied by Hastie *et al*.^24^ and Bailey *et al*.^25^. Our specific objectives were to (1) determine the relative importance that environmental cycles had on the presence of dolphins, (2) explore possible interactions between them, (3) assess whether patterns were consistent in time and space, and (4) explore the potential implications of the results for the conservation and management of this population of dolphins. Our main hypothesis was that environmental cycles would have site specific effects on the presence of bottlenose dolphins. Drawing upon the findings of the previous studies at individual sites, we predicted that our results would exhibit spatial variation in temporal patterns of occurrence, and that the relative importance of different environmental cycles would differ between sites. To do so, we used echolocation detections (CPOD; Chelonia Ltd.) coupled with circular statistics (Rayleigh test)^30^ and generalized additive mixed models (GAMM)^31^ to assess fine-scale temporal patterns of dolphin presence. We studied three discrete sites (Sutors, Chanonry and Spey Bay) which have been identified as important sites for this population in several studies^32–34^ and which differ in their physiographic characteristics such as tidal regime and bathymetry.

## Results

Deployments were made almost continuously at all three study sites during the 6-year study period, providing data with only a few gaps due to battery or equipment failure. The maximum number of clicks was reached in a very small proportion (<0.11%) of minutes, meaning that the CPODs were sampling for dolphin presence >99.8% of the time in all sites. In total, datasets included more than 23000 encounter-positive hours (Tables 1 and S1 in Supplementary Material). The highest number of encounters was recorded in Sutors (n = 15188), followed by Chanonry (n = 8406), with smaller numbers in Spey Bay (n = 4316).

**Table 1:** Summary of acoustic data obtained from 2010 to 2016.

	Sutors	Chanonry	Spey Bay
Location	57°41′25.02″N	57°35′9.36″N	57°41′23.53″N
	3°58′51.30″W	4°5′53.40 W	3°5′36.06″W
Hours recorded	49028	44445	47448
% time max. num. clicks reached	0.104%	0.001%	0.062%
Total number of encounters	15 188	8406	4316
Total encounter positive hours	12 516	7038	3749

### CPOD validation

Using PAMGuard click detections as a baseline, CPODs predicted dolphin presence with high accuracy at both filter settings: High-Moderate (98%) and High quality (100%) whereas their ability to predict dolphin absence was lower, 71% and 66% for High-Moderate and High quality trains respectively (Supplementary Material Table S2). The false positive rate was very low (0–1%) and the true negative rate was very high (100–98%).

Overall, CPODs therefore produced fewer dolphin detection positive hours than PAMGuard when using either train quality filter setting. Despite this, both PAMGuard and CPODs detected the same diel pattern over the whole deployment (Supplementary Material Fig. S1).

### Effect of each environmental cycle

In Sutors the number of encounters exceeded 25 in most months, allowing the Rayleigh test to be performed for almost the whole period. In Chanonry and Spey Bay, the number of encounters was sometimes fewer than 25 per month, especially in winter, so the Rayleigh test could not be performed in all cases.

**Figure 1:**
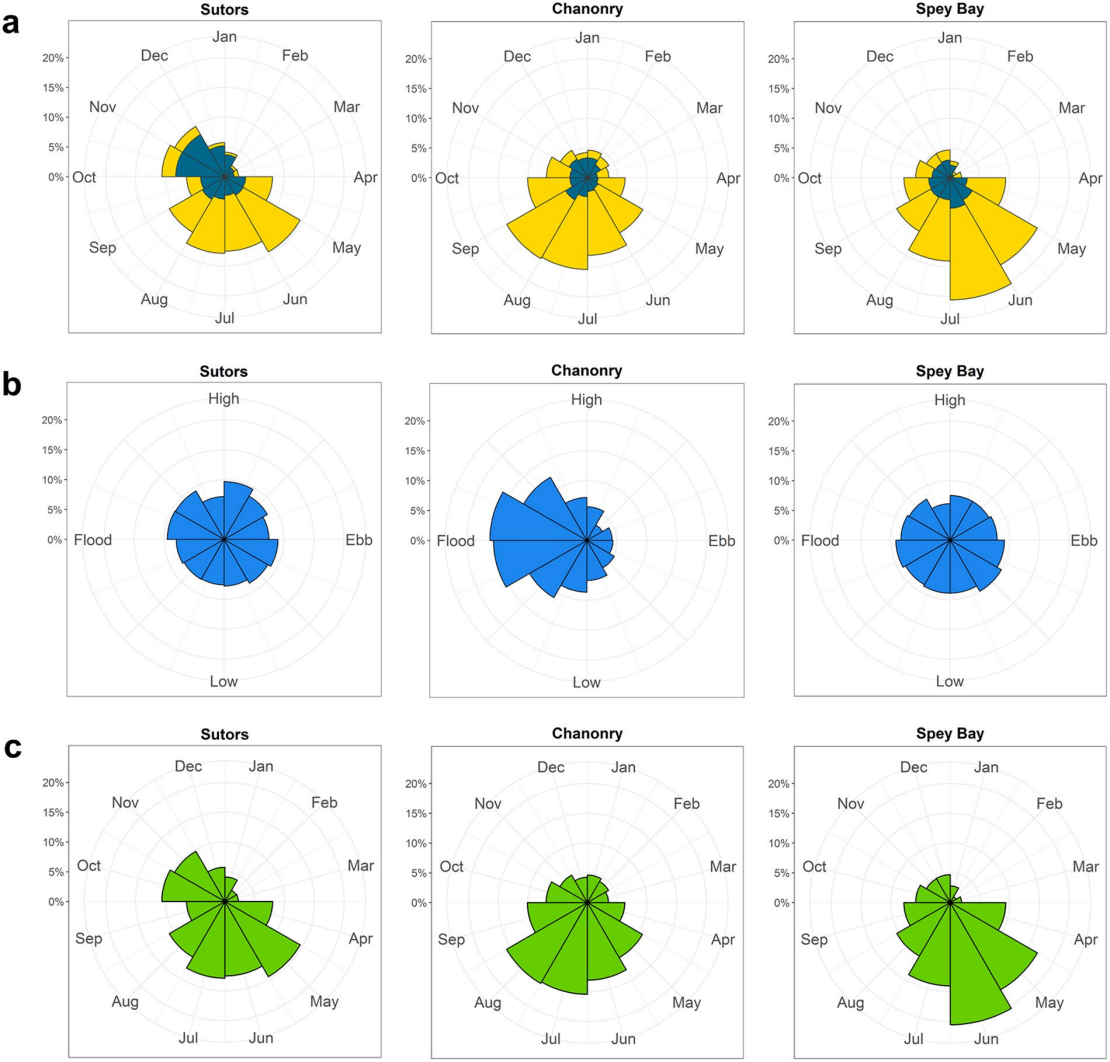
Proportion of encounters relative to the (**a**) diel cycle (yellow: encounters during the day; dark blue: encounters during the night), (**b**) tidal cycle and (**c**) seasonal cycle at the three sampling sites for the whole period studied (2010–2016).

**Figure 2:**
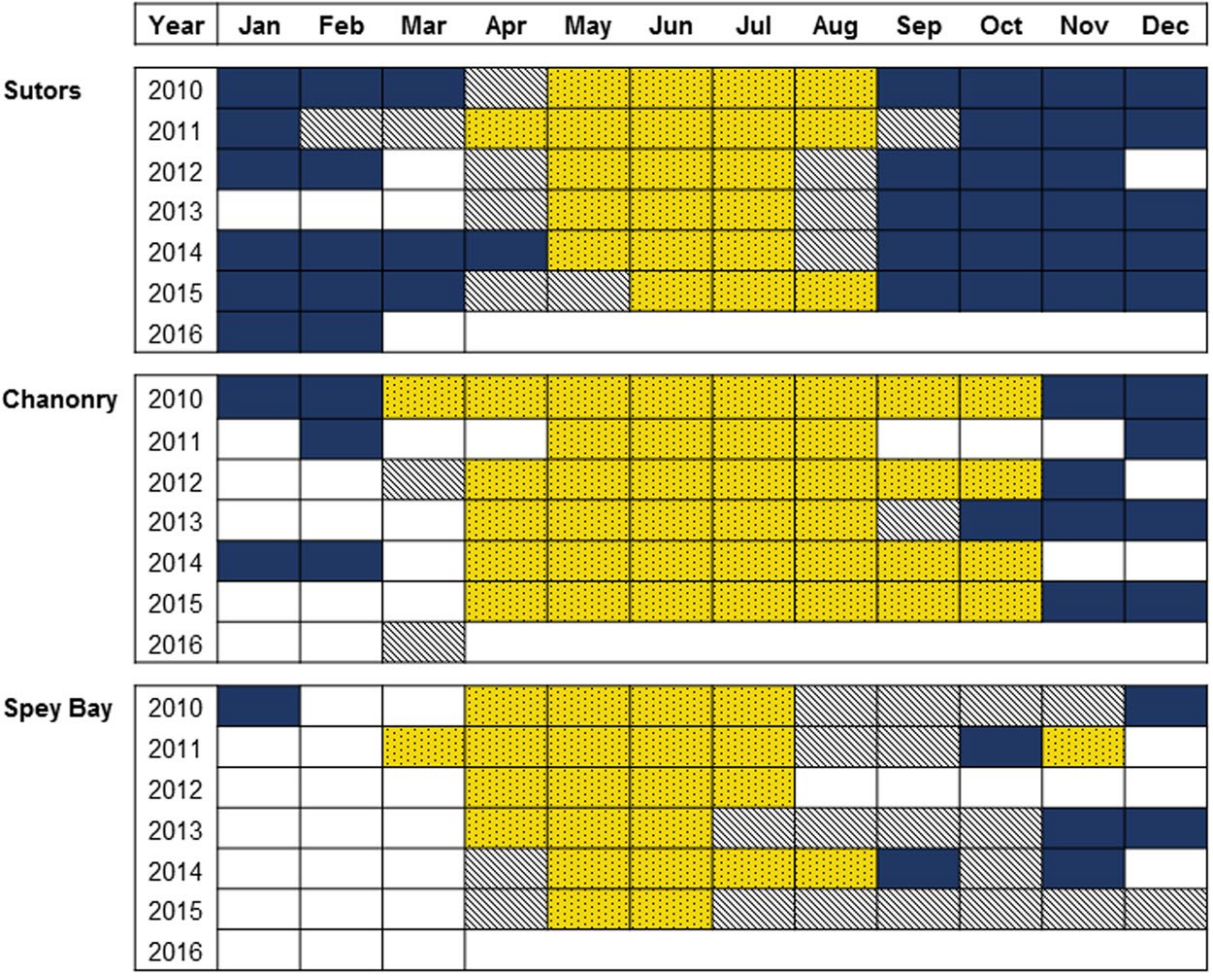
Months in which significant Rayleigh tests indicated that dolphin encounters were more likely in daytime are shown in yellow, and those in which encounters were more likely at night are shown in dark blue. Those months in which Rayleigh tests were not significant (P > 0.05) are shown with diagonal shading and white cells represent months with fewer than 25 encounters.

The relationship between the diel cycle and the occurrence of dolphins differed between sites (Fig. 1a). In Sutors and Chanonry, times of encounters were significantly different from a uniform distribution over the diel cycle for most months (Fig. 2). At these two sites, encounters most often occurred during daytime in summer and during night-time in autumn and early winter (circular mean vector during day and night respectively, see Table S3 in Supplementary Material for detailed information). In Spey Bay, fewer months were statistically significant and the Raleigh test provided no evidence for the seasonal variation in diel pattern seen at the other two sites.

**Figure 3:**
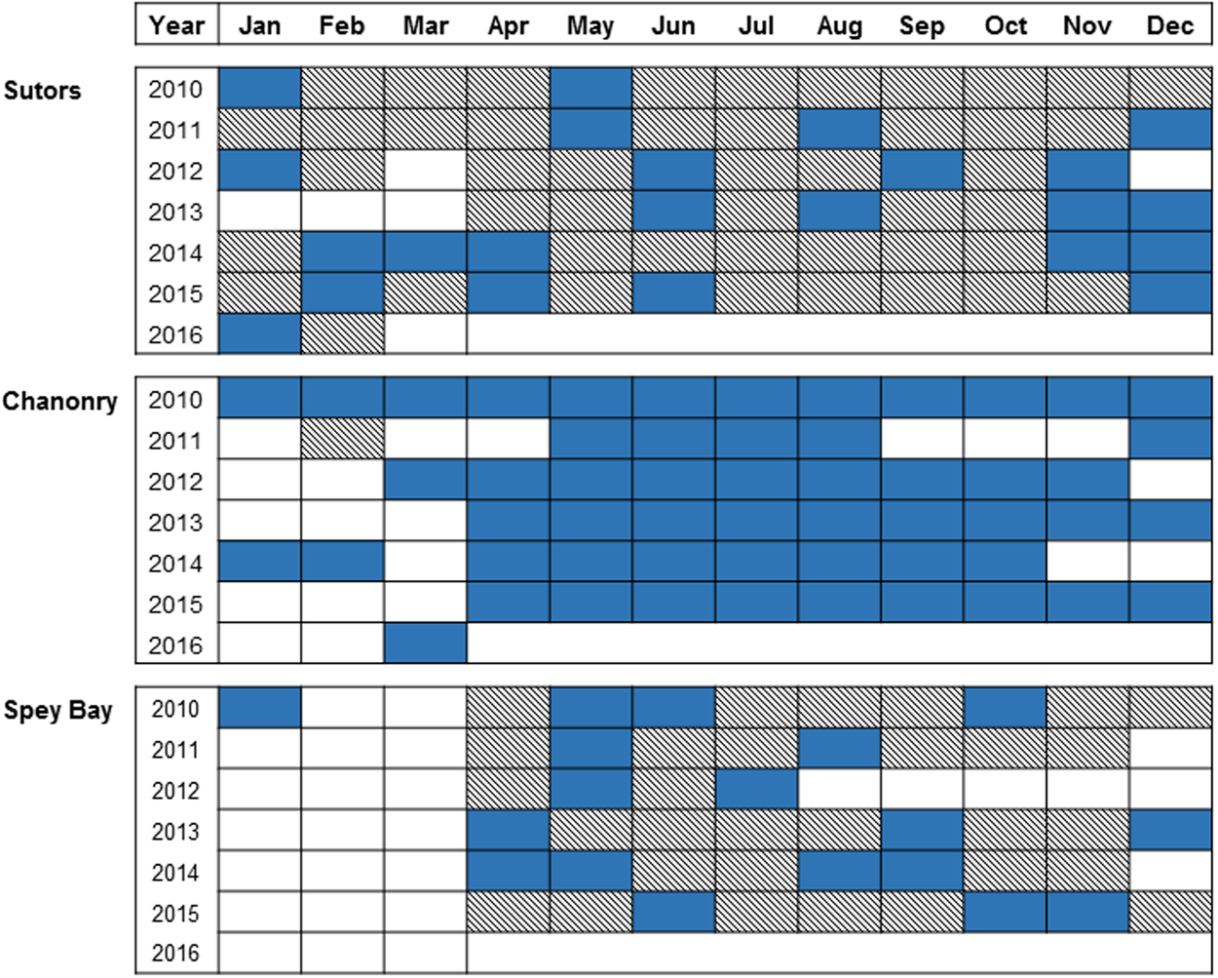
Months in which significant Rayleigh tests indicated that dolphin encounters were related to the tidal cycle, shown in blue. Those months in which Rayleigh tests were not significant (P > 0.05) are shown with diagonal shading and white cells represent months with fewer than 25 encounters.

The effect of the tidal cycle on the occurrence of dolphins also varied between sites. There was a strong relationship between dolphin presence and the tidal cycle at Chanonry (Fig. 3), where a high proportion of encounters occurred during the flood tide (Fig. 1b). Although some months showed a relationship with tidal cycle at the other sites (Fig. 3), occurrence did not peak consistently at any particular stage of the tide (Fig. 1b and Table S4 in Supplementary Material).

Encounters were distributed significantly differently from a uniform distribution over the seasonal cycle in all years at all sites (Rayleigh test: P < 0.01) with most encounters occurring during summer months (circular mean vector during summer; Table 2). Overall, mean length values (R) were slightly lower in Sutors than in the other two sites. Thus, while the highest proportion of encounters occurred in summer in all sites (Fig. 1c), seasonality was stronger in Chanonry and Spey Bay. In Sutors, the proportion of encounters remained high in autumn.

**Table 2:** Rayleigh Test results for the seasonal cycle in the three sites between 2010 and 2015.

	Sutors			Chanonry			Spey Bay		
	p	Mean	R	p	Mean	R	p	Mean	R
2010	<0.0001	July	0.238	<0.0001	June	0.166	<0.0001	June	0.227
2011	<0.0001	July	0.239	<0.0001	June	0.545	<0.0001	July	0.486
2012	<0.0001	July	0.403	<0.0001	July	0.515	—	—	—
2013	<0.0001	July	0.486	<0.0001	Aug	0.458	<0.0001	July	0.576
2014	<0.0001	July	0.216	<0.0001	July	0.351	<0.0001	July	0.482
2015	<0.0001	July	0.279	<0.0001	Aug	0.431	<0.0001	June	0.370

The Mean value shows the month in which the peak of dolphin encounters occurred. R is the length of the mean vector which provides an indication of the strength of the relationship.

### Interactions between cycles and their relative importance

The relative importance of each of the cycles varied among sites (Table 3). Diel and seasonal cycles were statistically significant at all sites. The tidal cycle was significant in Chanonry and Sutors but not in Spey Bay. The only interaction to have an effect on the presence of dolphin encounters was the interaction between diel and seasonal cycles. Based on the change in AIC, the most important covariates were the interaction diel*seasonal in Sutors, *tidal* in Chanonry and *seasonal* in Spey Bay.

**Table 3:** Summary results for the GAMM using tensor products (ti) smooths.

Covariate	Sutors				Chanonry				Spey Bay			
	Chi-sq	P	ΔAIC	AIC Rank	Chi-Sq	P	ΔAIC	AIC Rank	Chi-sq	P	ΔAIC	AIC Rank
*Diel*	617.2	<0.0001	−26.4	5	327.2	<0.0001	−200.9	3	187.5	<0.0001	−163.0	2
*Tidal*	98.9	<0.0001	−85.9	3	1307.3	<0.0001	−1333.9	1	- NS -			
*Seasonal*	397.3	<0.0001	−361.4	2	368.8	<0.0001	−36.2	5	198.9	<0.0001	−175.2	1
Diel*Seasonal	461726.1	<0.0001	−658.4	1	863.1	<0.0001	−200.0	4	39660.4	0.007	−37.9	4
*Year*			−74.4	4			−292.6	2			−55.1	3

ΔAIC: variation in the AIC value by removing the covariate. AIC Rank: relative importance of each covariate. Covariates ranked from 1 (highest AIC weight) to 5 (lowest).

Final GAMM models included *tidal*, the full tensor product diel*seasonal and *year* in Sutors and Chanonry. *Tidal* was not retained in Spey Bay. Both GAMM approaches, using temporal correlation corAr1 and gamm4, gave similar results (summaries included in the Supplementary Material).

**Figure 4:**
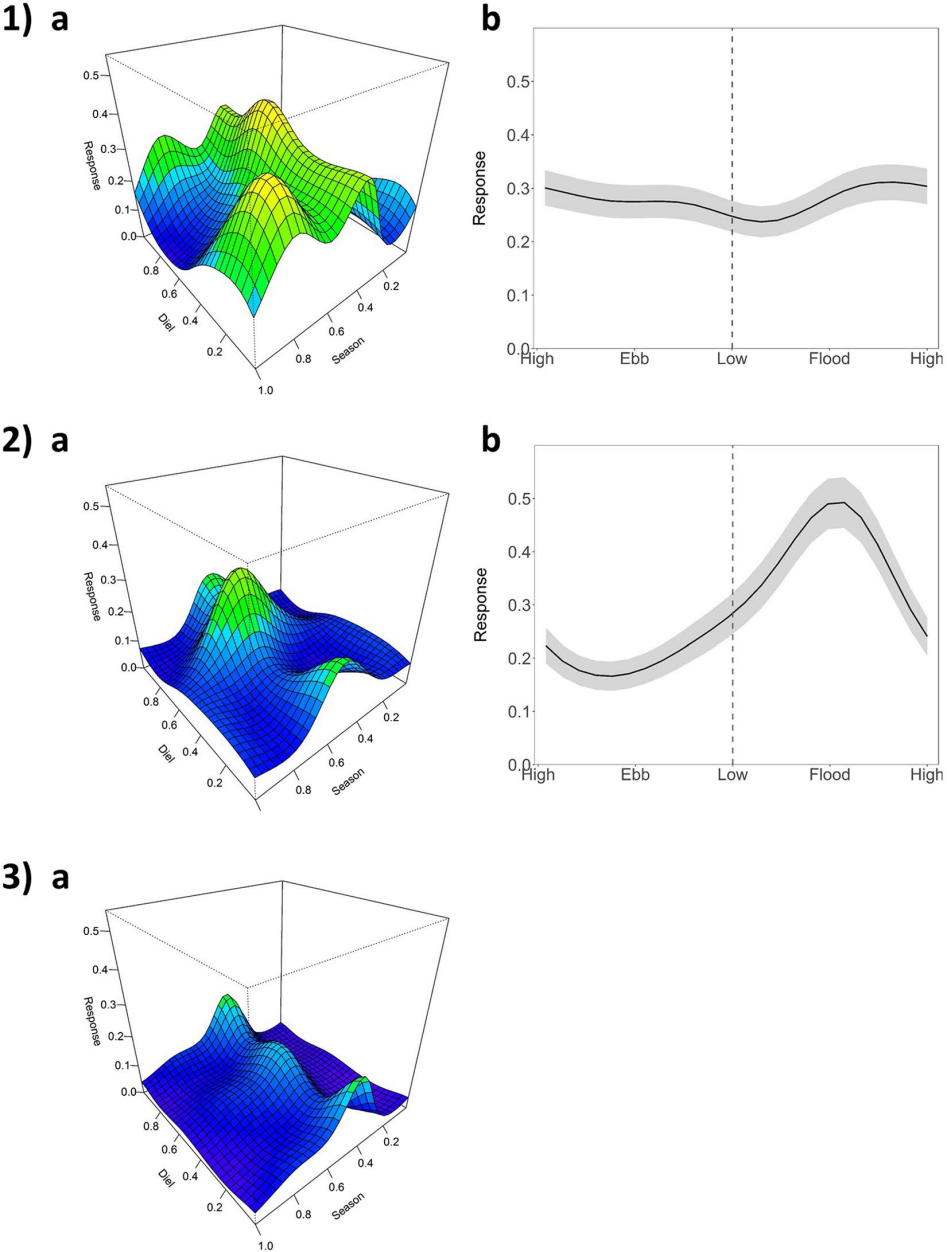
GAMM modelling results for (1) Sutors, (2) Chanonry and (3) Spey Bay. Representation on the response scale of the effect of the (**a**) interaction diel*seasonal (**b**) tidal variable (95% confidence intervals represented by grey areas) on the probability of dolphin encounters.

Dolphin encounters showed site-specific relationships with each of the temporal cycles and results matched patterns previously observed with circular statistics (Fig. 4). In Sutors, the diel behaviour was diurnal in summer and nocturnal in autumn. At this site, the probability of encounters remained high all day long during summer, with the highest probability during daytime. By the end of the year, around October-November, the situation changed and the probability of encounters was high only during night-time. In Chanonry the diel behaviour was diurnal during summer months with a higher probability of encounters during day-time. During the rest of the year the diel pattern was not clear but the probability was slightly higher at night. In Spey Bay a diel pattern was only evident during summer months. There the diel behaviour was crepuscular, with a higher probability of encounters around sunrise and sunset.

The importance of the tidal cycle was clear in Chanonry with the highest probability of encounters concentrated around the flood stage of the tide (between the low and high tide). In Sutors, although the variable was significant, the tidal influence was much weaker, with only a slight reduction in use after low tide and a small peak before high tide (Fig. 4c). As stated before, this variable was not significant in Spey Bay.

The probability of encounters showed the highest values during summer months at all sites. Seasonality was more evident in Chanonry and Spey Bay, with the highest values concentrated around the middle of the year. In Sutors, values remained higher than in the other two sites during the whole year, but especially around October-November.

Temporal correlation issues were corrected using both GAMM approaches, GAMM4 and temporal correlation corAr1 (Fig. S2 in Supplementary Material: example of ACF plots). No collinearity was found between the variables included in the final models.

## Discussion

Our analyses of changes in acoustic detections allowed us to compare the relative importance of tidal and diel cycles in the occurrence of dolphins at our three different sampling sites. Cetaceans are considered less likely to be influenced by tides in open coastal areas than in narrow channels^35,36^. In line with this, there was no effect of the tidal cycle at the most open of our study sites in Spey Bay (Fig. 3). In contrast, occurrence at Chanonry was strongly influenced by tide in all months of the year (Fig. 3). This site was within a narrow channel and subject to strong tidal flows which are known to influence fine-scale spatial movements of dolphins in this area^37^. Our acoustic data extended the results of previous visual surveys^25^ by confirming that this pattern dominated during both day and night. The Sutors site was within a similar, albeit slightly wider, channel, where previous visual studies had not detected strong tidal effects^24^. Our results show that the amplitude of the tide effect on dolphin activity is smaller than at Chanonry overall (Fig. 4a), yet the differences are highly significant and the pattern of use is clearly qualitatively different at the latter site, despite apparent similarities in the physiography of these two sites. Similarly, Pirotta *et al*. (2014) found that a tidal mixing parameter and tidal speed had a stronger influence on foraging behaviour in Chanonry than in Sutors^34^. Together, these studies highlight more subtle differences in the oceanography and/or prey populations at these sites that warrant further investigation. The diel cycle showed marked differences between sites, with evidence of consistent diurnal, nocturnal and crepuscular patterns of behaviour through the study period (Fig. 4). At a larger scale, Castellote *et al*. (2015) also found contrasting diel patterns in occurrence of bottlenose dolphins within Mediterranean MPAs located 90 km apart^38^. Our results highlight that site-specific variability in both tidal and diel behaviour persists at much smaller spatial scales (13.5 km).

By considering interactions between temporal variables, we revealed a consistent seasonal shift in diel behaviour, with dolphin occurrence within these inshore sites becoming highly nocturnal in autumn. This is particularly seen in Sutors, as the overall use of the other two sites is considerably reduced outside summer. As far as we know, this is the first time that a seasonal shift in diel presence has been documented in a resident population of marine mammals. Many authors have suggested that the cyclical behaviour of marine mammals is related to the avoidance of their predators or to the cyclicity of their prey^39–41^, but obtaining direct evidence to test these alternative hypotheses remains challenging. Although Orca (*Orcinus orca*) occur occasionally in this area^42^, there is no evidence of this dolphin population being regularly exposed to natural predators. Whilst predator avoidance behaviours may also occur in response to anthropogenic disturbance^43^, there is no evidence of a similar seasonal shift in diel patterns of any human activity that could elicit the patterns observed in our data. In contrast, the importance of these sites has been linked to foraging in the past^34,44^, suggesting that observed changes in occurrence are most likely related to differences in prey distribution^45^, prey density^46^, prey behaviour^39^ or prey catchability^47^. There are limited data on temporal and spatial variation in potential prey in the Moray Firth^48,49^, but no recent data that can be used to assess spatial variation at a scale relevant to our three study sites. Nevertheless, older data on fisheries and marine mammal predator-prey interactions might provide insights into the drivers of the cyclic patterns observed in this study. The Moray Firth had an important fishery for clupeids that overwintered in the area^50^ and in the late 1980s it was shown that these winter fish stocks were important for harbour seals. Pierce *et al*. (1991) discovered that harbour seals performed a seasonal shift in their diet and targeted clupeids in winter^51^. Harbour seals are benthic foragers^52^ and Thompson *et al*. (1991) linked the daily vertical migration of clupeids to the diel behaviour of seals which appeared to forage in daytime while fish were in tight schools in the deep channels of the inner Moray Firth^53^. In later years, clupeid stocks declined in the area and inter-annual variation in seal diet and physiology was found^54^. The variation in dolphin use of these same areas might be due to a recent recovery of fish stocks. We hypothesise that an influx of clupeids could be the driver of the diel behaviour of dolphins observed in autumn. Bottlenose dolphins may be using the deep channels to forage at night when the fish are closer to the surface, a foraging strategy that may be energetically more efficient^55^. However, studies of predator-prey behaviour would be needed in the area to prove this theory.

The seasonal cycle dominated patterns of occurrence at all sites, with a greater proportion of encounters in summer (Figs 1c and 4). These results confirm those observed in earlier boat-based visual studies of this population^24–26^. However, our results suggest that previous visual studies could have underestimated the importance of night-time use at the most intensively used of these sites (Sutors, Fig. 4a). In our study, acoustic data collected throughout the 24 hr cycle showed that there was a high probability of detection during night time in autumn. This highlights how acoustic studies may help overcome the restriction of visual surveys to daylight hours. At the same time, it is important to recognise that CPOD detection rates may be influenced by dolphin behaviour^56^. Marine mammal click beams are highly directional^57^ so that only clicks directed towards the acoustic device have the potential to be detected. Additionally, bottlenose dolphins echolocate mainly when traveling and foraging, and whistle when socialising^58,59^. Our comparison of CPOD detections and analysis of simultaneous broadband recordings support previous studies that demonstrate that CPODs provide a robust and conservative measure of dolphin occurrence within a range of almost 1000 m^27,60,61^. However, this comparison also highlighted that click absences should always be interpreted cautiously because the use of this conservative metric means that these will include some false negatives, and could also represent either an absence of animals or a change in acoustic behaviour. Broad band acoustic devices that detect other vocalisations^62^ could be used to explore this issue further. In the meantime, we assume that the most likely cause of observed changes in click detections is a change in dolphin occurrence, although we cannot rule out the possibility that this could reflect some other behavioural change.

This study highlights the importance of understanding temporal variation in behaviour to underpin more dynamic management of Marine Protected Areas. Although nocturnal activity has been detected in many studies of these animals^23,38,63^, visual methods are still widely used to study their distribution^64,65^. Visual surveys only provide data during daylight hours and may be underestimating the importance of certain locations during the night^23^. For example, our Sutors and Chanonry study sites are two of the most important areas for bottlenose dolphins within the Special Area of Conservation (SAC) that was established to protect their population^26,44^. However, these areas are also subject to a wide range of human activities, including vessel traffic and spoil dumping^66,67^. Previous studies have recognised the importance of these areas^26,32^, but the visual sightings used to underpin that mitigation work were focused on the summer months^68^. As result, some activities such as spoil dumping have been encouraged in autumn, when our results indicate that animals continue to use some of these sites albeit nocturnally.

In summary, this study presents evidence that environmental influences on dolphin behaviour can be highly site-specific, even at relatively fine spatial scales. These findings demonstrate the need to include dynamic environmental variables and interactions between them when modelling the habitat use of small cetaceans. Environmental cycles can interact with one another, producing the consistent seasonal shift in diel behaviour observed in this population. Similar patterns are likely to occur elsewhere but may have gone unnoticed. The challenge now is to develop a better understanding of the key drivers underpinning these patterns, and to identify how best to integrate this information into more effective marine management measures.

## Methods

### Study Area

The Moray Firth is a large coastal embayment on the East Coast of Scotland, which contains a Special Area of Conservation (SAC) that was designated for its resident population of bottlenose dolphins under the EU Habitats Directive (92/43/EEC)^69^.

**Figure 5:**
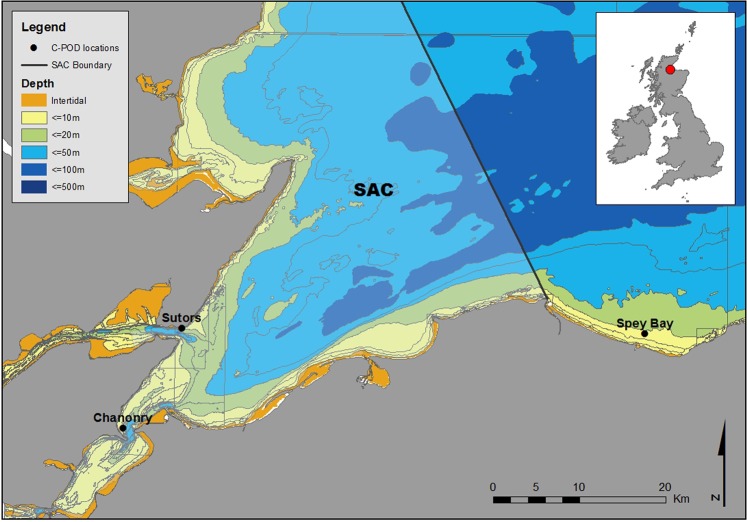
Map of the Moray Firth and the three sites studied: Sutors, Chanonry and Spey Bay. Detailed bathymetry and aerial photographs of the three sites are provided in Supplementary Material (Fig. S3).

Three discrete sites were studied: Sutors (57°41.41′N, 03°59.18′W), Chanonry (57°5.14′N, 04°5.85′W) and Spey Bay (57°41.32′N, 03°5.14′W) (Figs 5 and S3 in Supplementary Material). All three sites are in the core area of this bottlenose dolphin population’s distribution, where the presence of other species of dolphins is rare^70^. Sutors and Chanonry are constricted channels in the inner part of the firth that are subject to strong tidal currents and are used intensively by this population^26,32^. Spey Bay, located on the southern coast of the outer Moray Firth, has also been identified as an important area for this population^33^. It is a shallow coastal area with a low energy tidal regime^71^.

### Acoustic data collection

CPODs (Chelonia Ltd, UK) were used to provide data on temporal variation in the occurrence of dolphins at the three study sites. CPODs are acoustic data loggers, specifically designed to detect marine mammal echolocation clicks that have been widely used in recent years^34,72,73^. CPODs record data continuously on the time of occurrence of tonal clicks, as well as many other variables, within the frequency range 20 kHz to 160 kHz for up to 4 months. In this study, data were used from deployments made between January 2010 and March 2016. CPODs were moored in shallow waters (<13 m) using moorings with either a subsurface acoustic release or a surface buoy as described by Bailey *et al*.^27^. A target frequency of 50 kHz and a reference frequency of 70 kHz were used. The *scan limit* (maximum number of clicks recorded in each scan) was set to a maximum of 4096 clicks per minute to conserve memory.

### CPOD validation

CPOD performance within our study system was evaluated by comparing their detection rate to the one obtained through analyses of broadband recordings from SM2M devices (Wildlife Acoustics, MA, USA) using PAMGuard software^74^. This comparison used existing datasets generated in a previous study^75^, where the authors designed a conservative dolphin click classifier and a Gaussian mixture-model to identify buzzes (click trains associated with attempted prey captures). Here, those click and buzz detections were compared to data recorded by CPODs that had been deployed on the same mooring as the SM2M recorder between May and July of 2013. PAMGuard detections were considered to be an indicator of dolphins’ presence following the methodology described by Garrod *et al*.^61^.

### Environmental data

High and low tide times were obtained from the United Kingdom Hydrographic Office (www.gov.uk/ukho) using reference ports within 12 km of each sampling site (Port of Cromarty: 57°40′N, 04°00′W, for Sutors and Chanonry; Port of Lossiemouth: 57°43′N, 03°17′W, for Spey Bay). Sunrise and sunset times were calculated for each of the sites using the ‘maptools’ library^76^ in R^77^.

### Data analyses

Data from the CPODs were processed using the manufacturer’s CPOD software (www.chelonia.co.uk). As recommended by the manufacturer, only click trains classified as *High* and *Moderate Quality* were used in this study. The statistical programme R v. 3.3.1^77^ was used in all subsequent analyses.

**Figure 6:**
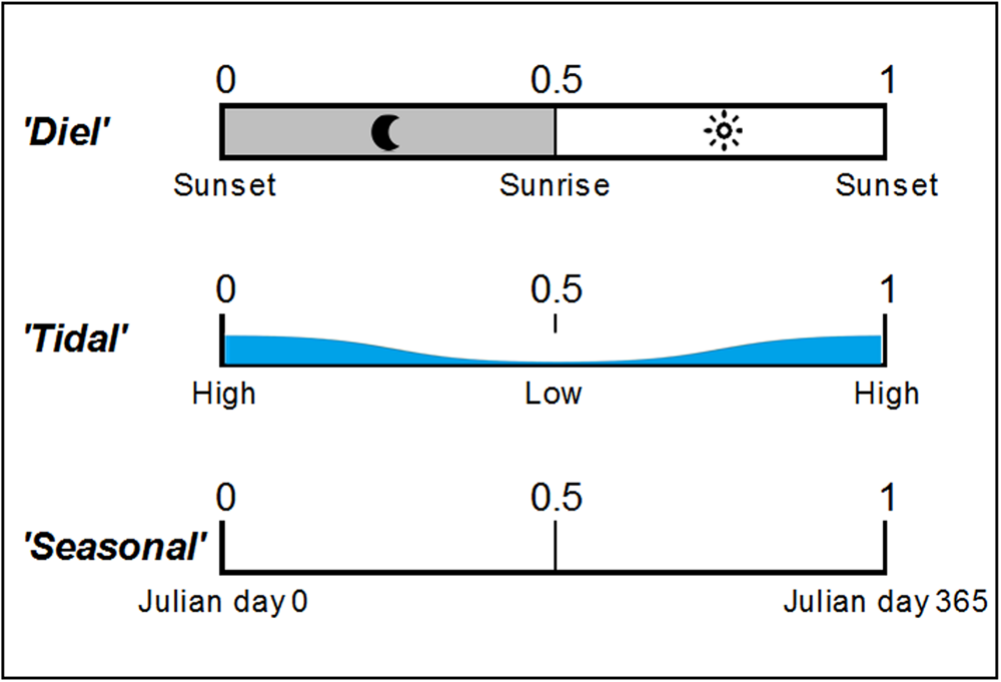
Schematic of the assignment of values for the diel, tidal and seasonal variables. All variables ranged from 0 to 1.

Individual click trains were grouped into encounters. Encounters were defined as groups of click trains at least 10 minutes apart^78,79^. Since the main focus was on studying rhythms, circular statistics were used to describe the effect of each of the environmental cycles. For this, the time of the mid-point of each encounter was transformed into three different cyclical indices derived from its position in each of the cycles (diel, tidal and seasonal). The *diel* variable was obtained by measuring the time difference between the encounter and the previous sunset. Due to the high latitude of the research area and the variability of day length across the year, its values were normalized: 0 and 1 corresponded to sunset whereas sunrise was fixed to 0.5, meaning that the time span between 0 and 0.5 varied as a function of the season^80^. The *tidal* variable was calculated following the same methodology: 0 and 1 corresponded to high tide and 0.5 to low tide. For the *seasonal* variable the 0 was set at Julian day 1 (Fig. 6). Rayleigh tests were performed to determine whether encounters were significantly clustered around a mean^30^. For diel and tidal cycles, tests were conducted on a monthly basis for each month with ≥25 encounters in order to increase the statistical power. For the seasonal cycle, analyses were conducted on a yearly basis. When the null hypotheses was rejected (P < 0.05), the mean vector (peak of dolphin presence) and mean resultant length (length of the mean vector, an indicator of the dispersion of the data) were calculated. Analyses were conducted using the R package ‘circular’^81^.

Generalized additive mixed models (GAMMs) were then used to explore non-linear effects of the diel, tidal and seasonal cycles on the presence of bottlenose dolphins. Presence/absence of dolphin encounters per hour was set as the response variable. *Diel*, *tidal* and *seasonal* were included as explanatory variables and defined by cyclic cubic regression splines. *Year* was introduced as a factor. All possible interactions were included in the models as tensor products (function ‘*ti’*), as recommended when the main effects and any lower interactions are also present^82^. The importance of each term was determined by removing them sequentially from the model^16^ and exploring the variation in the Akaike Information Criterion (AIC)^83^. Once the main interactions were determined for each of the sites, full tensor product smooths (function ‘*t2*’ and ‘*te’*) were used in the final models.

The binomial GAMMs above were fitted using the’gamm4’ library^84^. Because ‘gamm4’ does not allow accounting for the inherent temporal autocorrelation in the model residuals, the presence of dolphin encounters in the previous hour was included as a fixed effect in the models^85^. Finally, the robustness of all models were checked by also using the function ‘gamm’ in ‘the mgcv’ library^82^. This may in some cases be less stable for binomial data but does account for residual autocorrelation. In these models, temporal autocorrelation was assumed to be produced by an autoregressive process of order 1 (corAR1) within each day.

Data from the three sites were modelled separately to assess variability at each location and to avoid unnecessary complexity with site interactions. Multicollinearity between explanatory spline functions was checked using the concurvity function. Autocorrelation plots (ACF) and partial autocorrelation plots (pACF) were used to check the level of autocorrelation in residuals.

## Supplementary information

Supplementary Information

## Data Availability

The authors are in the process of uploading the data to Dryad.
