# Supplementary material for: Fine scale spatial variability in the influence of environmental cycles on the occurrence of dolphins at coastal sites
Source: Sci Rep. 2019 Feb 22;9:2548. doi: 10.1038/s41598-019-38900-4 (PMC6385188; doi:10.1038/s41598-019-38900-4)
Supplement: Supplementary file 1 — Supplementary Information [file 41598_2019_38900_MOESM1_ESM.pdf]

Fine scale spatial variability in the influence of environmental cycles on the occurrence of dolphins at coastal sites

Oihane Fernandez-Betelu, Isla M. Graham, Thomas Cornulier & Paul M. Thompson

SUPPLEMENTARY MATERIAL

Table S1: Total number of encounters per year and month at each site.

	Month												
	**1**	**2**	**3**	**4**	**5**	**6**	**7**	**8**	**9**	**10**	**11**	**12**	***Tot. Num. Encounters***
**Sutors**													
2010	69	58	108	352	419	380	348	331	202	360	388	146	*3161*
2011	73	35	84	261	380	300	292	298	81	271	193	228	*2496*
2012	130	50		128	464	377	332	275	161	303	165		*2385*
2013				101	332	335	382	230	172	181	216	135	*2084*
2014	193	75	56	184	403	292	359	255	204	275	271	154	*2721*
2015	59	70	60	165	202	232	294	246	159	217	243	210	*2157*
2016	123	32	22										*177*
**Chanonry**													
2010	185	170	159	99	179	158	256	219	168	99	34	74	*1800*
2011	24	46	15	1	116	187	151	146			17	43	*746*
2012	11	8	45	103	110	113	213	240	109	32	57	18	*1059*
2013				127	184	224	317	334	226	213	187	125	*1937*
2014	186	94	20	99	218	259	265	184	172	105	20		*1622*
2015			9	86	99	133	141	196	167	136	131	97	*1195*
2016			47										*47*
**Spey Bay**													
2010	68	6	7	66	110	129	51	36	25	60	63	26	*647*
2011	7	11	42	99	102	146	136	141	98	50	25	22	*879*
2012	5	12	7	48	112	103	82						*369*
2013				46	129	154	105	102	68	26	26	26	*682*
2014	13	15	3	68	158	157	151	110	101	79	48	22	*925*
2015	7	2	14	66	126	198	105	63	47	38	40	108	*814*
2016	22	2	12										*36*

Table S2: Results of PAMGUARD dolphin click and buzz detection and corresponding true positive (TP), false positive (FP), true negative (TN) and false negative (FN) hours and ratios and positive predictive (PPV) and negative predictive (NPV) values using High and High and Moderate quality trains from CPOD software. Following Garrod et al. (2018) methodology:

$$PPV = \frac{TP}{(TP + FP)}; \qquad NPV = \frac{TN}{(TN + FN)}$$

PAMGUARD				CPOD						
**Detection type**	**Total Effort (h)**	**Dolphin Present (h)**	**Dolphin Absent (h)**	**Filter**	**TP (h, TPR)**	**FP (h, FPR)**	**TN (h, TNR)**	**FN (h, FNR)**	**PPV**	**NPV**
Clicks	1215	957	258	Hi	458, 48%	0, 0%	257, 100%	499, 52%	100%	66%
				Hi-Mo	568, 59%	3, 1%	253, 98%	395, 41%	98%	71%
Buzzes	1215	714	501	High	440, 62%	19, 4%	482, 96%	274, 38%	94%	72%
				Hi-Mo	529, 74%	38, 8%	463, 92%	185, 26%	90%	78%

Table S3: Summary data table for the diel cycle. Bold: direction of the circular mean vector (0: Sunset, 180: Sunrise). In brackets: length of the mean vector which provides an indication of the strength of the relationship.

	Month											
	**1**	**2**	**3**	**4**	**5**	**6**	**7**	**8**	**9**	**10**	**11**	**12**
**Sutors**												
2010	**75.7** (0.5)	**57.2** (0.3)	**49.4** (0.3)		**280.3** (0.3)	**268.4** (0.3)	**273.1** (0.3)	**270.5** (0.3)	**78.0** (0.2)	**65.1** (0.4)	**78.0** (0.5)	**79.2** (0.6)
2011	**87.9** (0.4)			**242.9** (0.3)	**256.0** (0.3)	**289.9** (0.3)	**266.0** (0.2)	**302.0** (0.1)		**65.6** (0.4)	**64.7** (0.5)	**70.3** (0.6)
2012	**72.5** (0.6)	**72.3** (0.5)			**259.7** (0.4)	**252.1** (0.3)	**271.2** (0.2)		**64.9** (0.4)	**55.9** (0.5)	**71.4** (0.5)	
2013					**263.4** (0.4)	**263.2** (0.3)	**275.1** (0.2)		**62.4** (0.3)	**55.7** (0.5)	**80.0** (0.5)	**71.5** (0.6)
2014	**74.8** (0.6)	**85.2** (0.6)	**77.1** (0.5)	**91.6** (0.1)	**261.6** (0.3)	**255.6** (0.3)	**240.6** (0.3)		**51.6** (0.2)	**63.2** (0.4)	**62.7** (0.6)	**73.0** (0.6)
2015	**64.5** (0.6)	**93.9** (0.5)	**86.2** (0.3)			**271.5** (0.4)	**278.2** (0.2)	**264.6** (0.1)	**62.9** (0.4)	**76.4** (0.5)	**64.4** (0.4)	**77.3** (0.5)
2016	**68.8** (0.6)	**57.3** (0.7)										
**Chanonry**												
2010	**112.4** (0.4)	**120.9** (0.3)	**192.0** (0.2)	**287.0** (0.3)	**278.9** (0.6)	**270.0** (0.6)	**258.2** (0.4)	**264.1** (0.3)	**233.5** (0.4)	**231.7** (0.3)	**148.2** (0.4)	**78.5** (0.5)
2011		**113.2** (0.4)			**263.3** (0.4)	**265.6** (0.5)	**269.8** (0.5)	**247.5** (0.6)				**97.1** (0.4)
2012				**266.2** (0.5)	**311.9** (0.4)	**282.7** (0.3)	**254.1** (0.4)	**256.1** (0.4)	**238.9** (0.3)	**222.3** (0.5)	**106.7** (0.5)	
2013				**326.9** (0.2)	**262.5** (0.3)	**287.2** (0.3)	**272.6** (0.4)	**293.9** (0.1)		**154.8** (0.3)	**114.5** (0.3)	**84.5** (0.3)
2014	**89.9** (0.3)	**31.0** (0.2)		**290.6** (0.5)	**268.6** (0.3)	**262.1** (0.3)	**281.7** (0.3)	**228.7** (0.4)	**245.4** (0.4)	**215.4** (0.3)		
2015				**249.5** (0.3)	**266.2** (0.5)	**268.4** (0.6)	**261.6** (0.2)	**248.0** (0.3)	**254.2** (0.4)	**221.6** (0.3)	**146.6** (0.3)	**126.3** (0.4)
2016												
**Spey Bay**												
2010	**69.6** (0.5)			**305.2** (0.4)	**267.8** (0.2)	**283.0** (0.4)	**227.6** (0.4)					**51.7** (0.4)
2011			**279.8** (0.3)	**303.7** (0.3)	**291.4** (0.3)	**280.2** (0.2)	**263.1** (0.4)			**141.0** (0.3)	**195.2** (0.5)	
2012				**318.6** (0.3)	**327.1** (0.3)	**243.9** (0.4)	**246.0** (0.3)					
2013				**308.6** (0.4)	**273.4** (0.5)	**283.2** (0.2)					**65.6** (0.4)	**90.3** (0.3)
2014					**291.8** (0.3)	**285.4** (0.3)	**264.2** (0.2)	**271.1** (0.3)	**6.4** (0.3)		**79.3** (0.4)	
2015					**300.4** (0.4)	**264.8** (0.3)						
2016												

Table S4: Summary data table for the tidal cycle. Bold: direction of the circular mean vector (0: High tide, 180: Low tide). In brackets: R, length of the mean vector which provides an indication of the strength of the relationship.

	Month											
	1	2	3	4	5	6	7	8	9	10	11	12
**Sutors**												
2010	**81.7** (0.4)				**339.0** (0.1)							
2011					**350.1** (0.1)			**278.2** (0.1)				**12.8** (0.1)
2012	**340.9** (0.2)					**263.7** (0.1)			**13.1** (0.2)		**31.0** (0.2)	
2013						**268.8** (0.1)		**304.3** (0.1)			**36.4** (0.1)	**62.9** (0.2)
2014		**61.9** (0.2)	**7.7** (0.3)	**270.8** (0.2)							**30.2** (0.2)	**30.3** (0.2)
2015		**58.7** (0.2)		**293.5** (0.2)		**37.1** (0.1)						**59.6** (0.1)
2016	**94.4** (0.2)											
**Chanonry**												
2010	**249.9** (0.3)	**248.4** (0.4)	**263.9** (0.4)	**254.8** (0.5)	**301.1** (0.4)	**263.3** (0.3)	**259.5** (0.3)	**262.8** (0.3)	**262.3** (0.4)	**263.2** (0.5)	**271.0** (0.7)	**274.3** (0.6)
2011					**263.1** (0.4)	**237.8** (0.2)	**254.7** (0.4)	**239.9** (0.4)				**281.9** (0.5)
2012			**261.1** (0.5)	**267.0** (0.5)	**289.1** (0.5)	**246.3** (0.3)	**262.1** (0.4)	**254.4** (0.3)	**263.0** (0.5)	**262.1** (0.4)	**279.3** (0.4)	
2013				**263.5** (0.4)	**245.6** (0.4)	**255.2** (0.4)	**246.8** (0.2)	**248.3** (0.2)	**262.1** (0.3)	**278.2** (0.3)	**292.8** (0.3)	**278.1** (0.4)
2014	**245.5** (0.3)	**250.5** (0.2)		**249.1** (0.2)	**232.9** (0.4)	**254.7** (0.3)	**264.8** (0.4)	**263.6** (0.4)	**259.7** (0.3)	**271.4** (0.4)		
2015				**298.9** (0.5)	**322.2** (0.4)	**285.2** (0.4)	**265.1** (0.5)	**260.5** (0.4)	**280.7** (0.4)	**265.9** (0.3)	**283.1** (0.5)	**285.9** (0.5)
2016			**309.6** (0.3)									
**Spey Bay**												
2010	**270.1** (0.3)				**122.3** (0.3)	**33.5** (0.2)				**101.9** (0.2)		
2011					**172.9** (0.2)			**163.3** (0.2)				
2012					**164.5** (0.3)		**201.3** (0.2)					
2013				**99.9** (0.3)					**184.7** (0.3)			**252.2** (0.4)
2014				**239.8** (0.3)	**177.0** (0.2)			**3.2** (0.2)	**138.5** (0.2)			
2015						**233.3** (0.2)				**204.7** (0.4)	**207.4** (0.3)	
2016												

Figure S1: Cumulative detection positive hours for both SM2M and CPODs for each hour of the day during the whole deployment. Cross: SM2M click detections; Solid square: SM2M buzz detections; Solid triangle: CPOD detections High-Moderate quality trains; Solid circle: CPOD detections High quality trains.

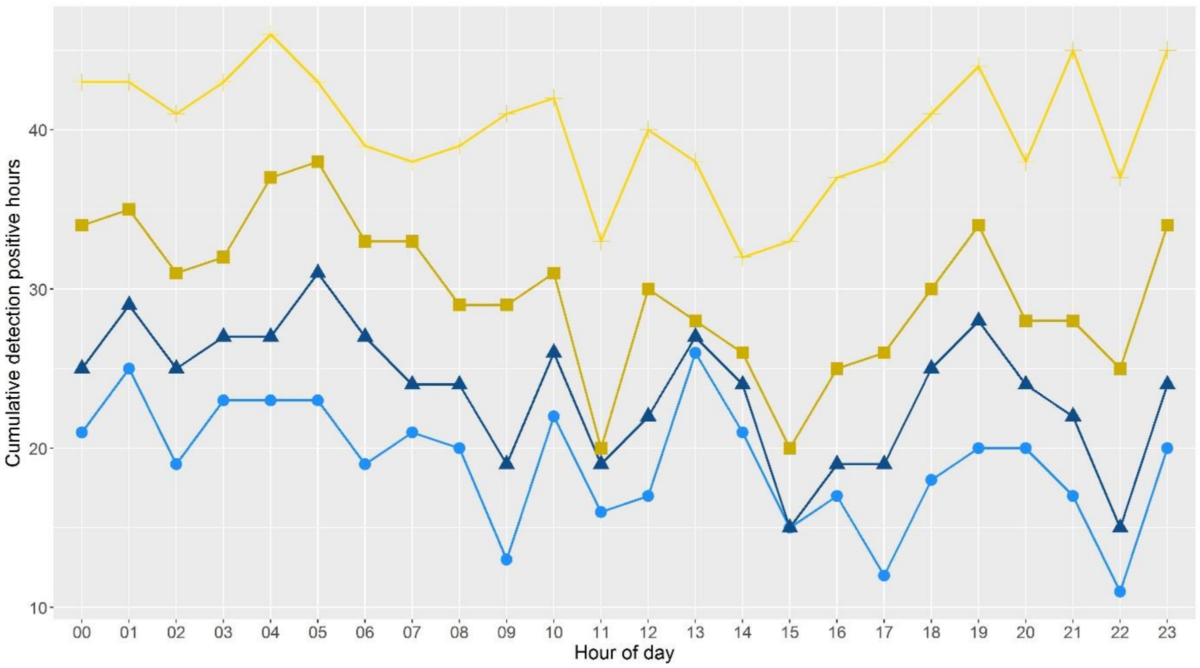

Figure S2: ACF plots for the GAMM models in Sutors.

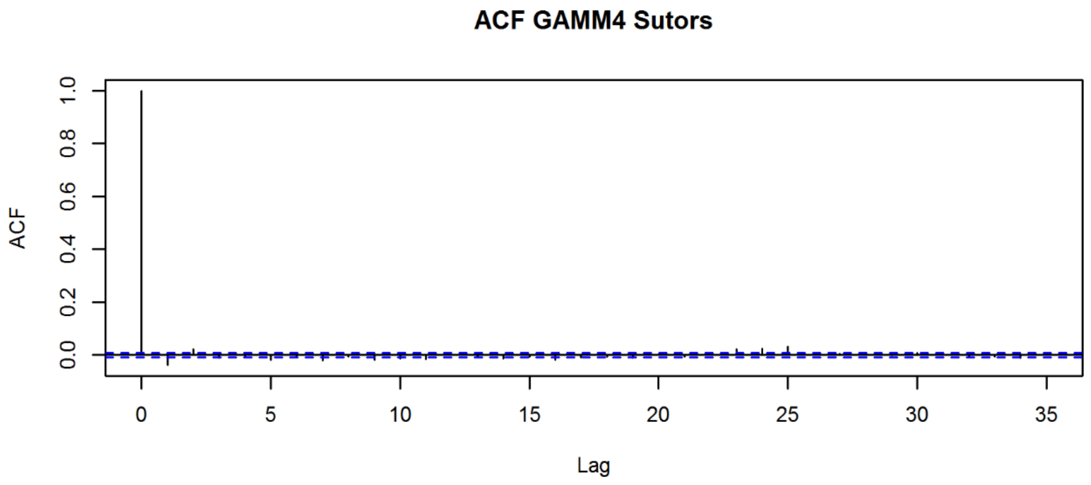

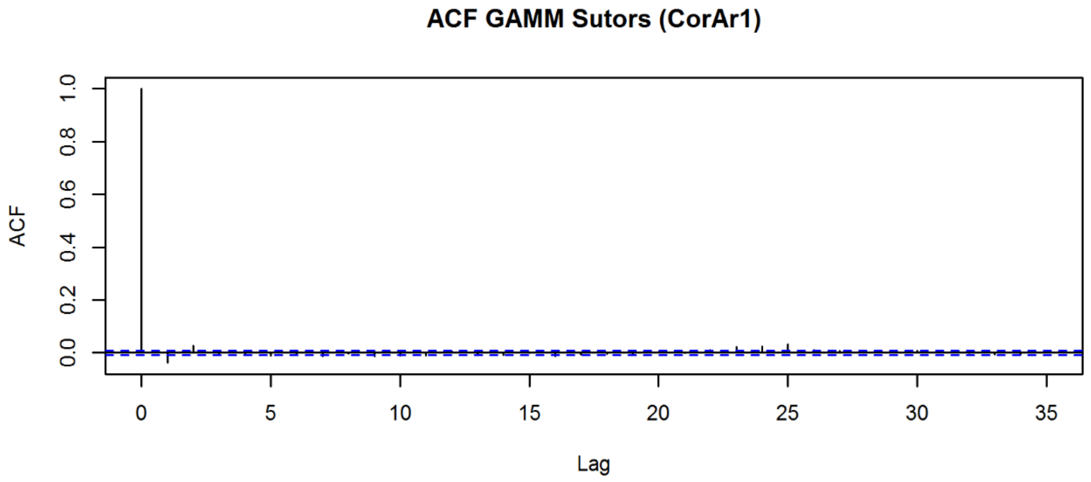

Figure S3: Detailed bathymetry and aerial pictures of the study sites. Top right and middle right images retrieved from CANMORE National Record of the Historic Environment - © Crown Copyright: Historic Environment Scotland. Bottom right image belongs to the University of Aberdeen.

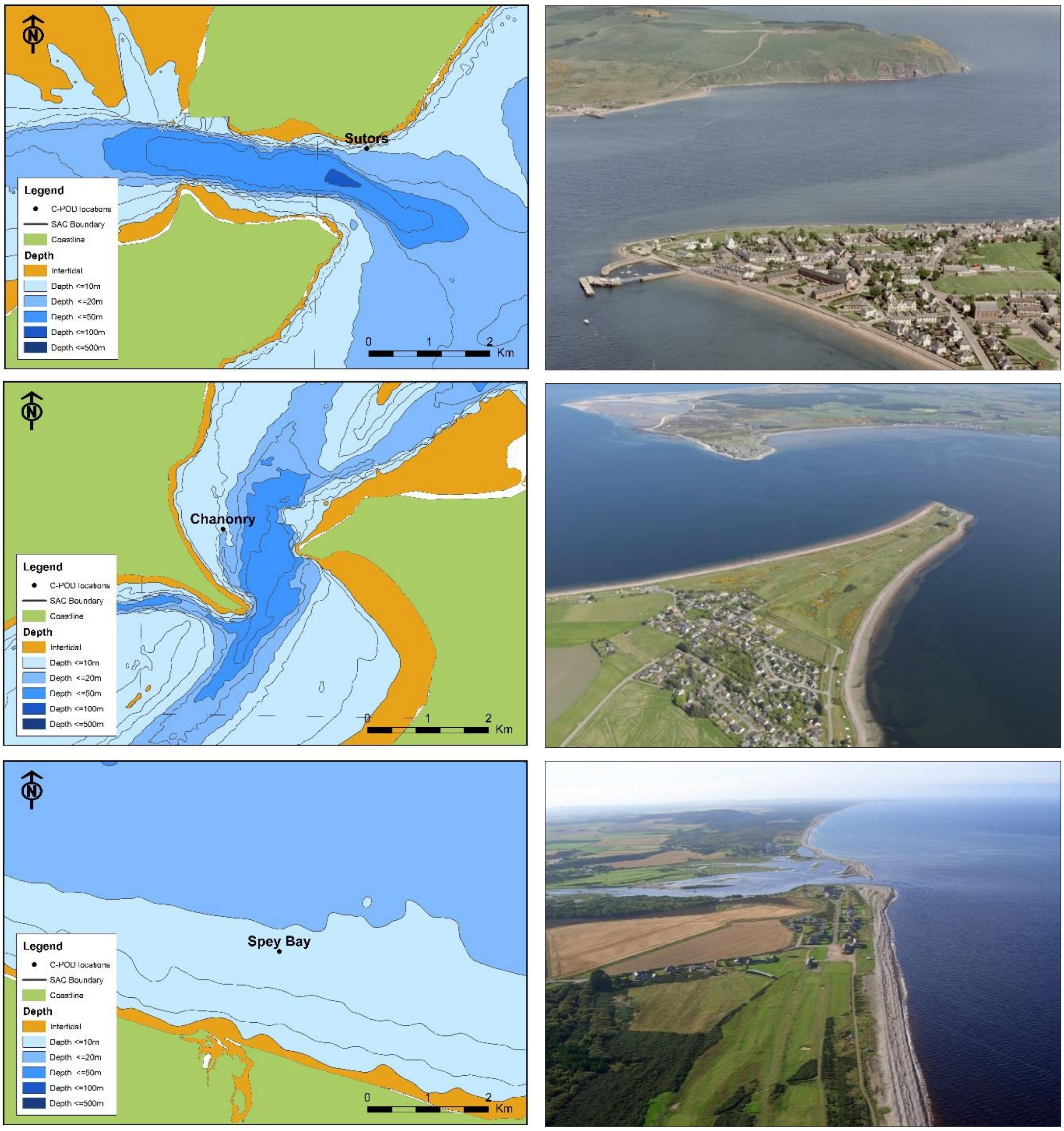

Summary of GAMM models for each site

```
> summary(gamm.G4.SUTORS$gam)

Family: binomial
Link function: logit

Formula:
presence ~ s(tidal, bs = "cc", k = 6) + t2(diel, seasonal, bs = "cc", k = 6) + PresLag1 + year

Parametric coefficients:
            Estimate Std. Error z value Pr(>|z|)
(Intercept)  -1.32749    0.04133 -32.120  < 2e-16 ***
PresLag1      0.80727    0.02456  32.864  < 2e-16 ***
year2011     -0.26510    0.05794  -4.576 4.75e-06 ***
year2012     -0.10299    0.06152  -1.674   0.0941 .
year2013     -0.36845    0.06195  -5.948 2.72e-09 ***
year2014     -0.14470    0.05736  -2.523   0.0116 *
year2015     -0.42969    0.05826  -7.375 1.65e-13 ***
year2016     -0.49917    0.12517  -3.988 6.67e-05 ***
---
Signif. codes:  0 '***' 0.001 '**' 0.01 '*' 0.05 '.' 0.1 ' ' 1

Approximate significance of smooth terms:
                       edf Ref.df  Chi.sq p-value
s(tidal)             3.748      4    99.4  <2e-16 ***
t2(diel,seasonal)   19.892     24 21765.1  <2e-16 ***
---
Signif. codes:  0 '***' 0.001 '**' 0.01 '*' 0.05 '.' 0.1 ' ' 1

R-sq.(adj) =  0.127
glmer.ML =  45785  Scale est. = 1         n = 49002


> summary(gamm.Ar1.SUTORS$gam)

Family: binomial
Link function: logit

Formula:
```

```
presence ~ s(tidal, bs = "cc", k = 6) + te(diel, seasonal, bs = "cc", k = 6) + year

Parametric coefficients:
            Estimate Std. Error t value Pr(>|t|)
(Intercept)  -1.02167   0.04430 -23.065  < 2e-16 ***
year2011    -0.29657   0.06348  -4.672 3.00e-06 ***
year2012    -0.11199   0.06738  -1.662  0.09648 .
year2013    -0.39851   0.06779  -5.879 4.15e-09 ***
year2014    -0.16116   0.06282  -2.565  0.01031 *
year2015    -0.47792   0.06390  -7.480 7.58e-14 ***
year2016    -0.52993   0.13781  -3.845  0.00012 ***
---
Signif. codes:  0 ‘***’ 0.001 ‘**’ 0.01 ‘*’ 0.05 ‘.’ 0.1 ‘ ’ 1

Approximate significance of smooth terms:
                       edf Ref.df     F p-value
s(tidal)             3.753      4 24.57  <2e-16 ***
te(diel,seasonal)   22.305     24 72.72  <2e-16 ***
---
Signif. codes:  0 ‘***’ 0.001 ‘**’ 0.01 ‘*’ 0.05 ‘.’ 0.1 ‘ ’ 1

R-sq.(adj) =  0.0864
Scale est. = 1         n = 49028


> summary(gamm.G4.CHANONRY$gam)

Family: binomial
Link function: logit

Formula:
presence ~ s(tidal, bs = "cc", k = 6) + t2(diel, seasonal, bs = "cc", k = 6) + PresLag1 + year

Parametric coefficients:
             Estimate Std. Error z value Pr(>|z|)
(Intercept)  -2.024386   0.044588 -45.402  < 2e-16 ***
PresLag1      0.954704   0.033104  28.840  < 2e-16 ***
year2011     -0.729255   0.074219  -9.826  < 2e-16 ***
year2012     -0.669164   0.066114 -10.121  < 2e-16 ***
year2013      0.294083   0.064757   4.541 5.59e-06 ***
```

```
year2014    -0.002885   0.064128  -0.045   0.9641
year2015    -0.289527   0.067373  -4.297 1.73e-05 ***
year2016    -0.384984   0.212035  -1.816   0.0694 .
---
Signif. codes:  0 ‘***’ 0.001 ‘**’ 0.01 ‘*’ 0.05 ‘.’ 0.1 ‘ ’ 1

Approximate significance of smooth terms:
                          edf Ref.df Chi.sq  p-value
s(tidal)                 3.936      4   1313  < 2e-16 ***
t2(diel,seasonal)    19.784     24   3503 3.05e-15 ***
---
Signif. codes:  0 ‘***’ 0.001 ‘**’ 0.01 ‘*’ 0.05 ‘.’ 0.1 ‘ ’ 1

R-sq.(adj) =  0.142
glmer.ML =  30682  Scale est. = 1         n = 44421



> summary(gamm.Ar1.CHANONRY$gam)

Family: binomial
Link function: logit

Formula:
presence ~ s(tidal, bs = "cc", k = 6) + te(diel, seasonalg, bs = "cc", k = 6) + year

Parametric coefficients:
            Estimate Std. Error t value Pr(>|t|)
(Intercept)   -1.744943   0.047512 -36.726  < 2e-16 ***
year2011    -0.761078   0.080742  -9.426  < 2e-16 ***
year2012    -0.694266   0.071834  -9.665  < 2e-16 ***
year2013     0.328248   0.069944   4.693 2.70e-06 ***
year2014     0.002181   0.069524   0.031   0.9750
year2015    -0.330034   0.073352  -4.499 6.83e-06 ***
year2016    -0.441989   0.234212  -1.887   0.0591 .
---
Signif. codes:  0 ‘***’ 0.001 ‘**’ 0.01 ‘*’ 0.05 ‘.’ 0.1 ‘ ’ 1
Approximate significance of smooth terms:
                          edf Ref.df      F p-value
s(tidal)                 3.938      4 314.98  <2e-16 ***
te(diel,seasonal)    22.334     24  46.57  <2e-16 ***
```

```
---
Signif. codes:  0 '***' 0.001 '**' 0.01 '*' 0.05 '.' 0.1 ' ' 1

R-sq.(adj) =  0.108
Scale est. = 1         n = 44445
```

```
> summary(gamm.G4.SPEYBAY$gam)

Family: binomial
Link function: logit

Formula:
presence ~ t2(diel, seasonal, bs = "cc", k = 6) + PresLag1 + year

Parametric coefficients:
            Estimate Std. Error z value Pr(>|z|)
(Intercept)  -3.21278    0.05828 -55.123  < 2e-16 ***
PresLag1      1.21758    0.04433  27.468  < 2e-16 ***
year2011      0.31057    0.07567   4.104 4.05e-05 ***
year2012     -0.07575    0.09651  -0.785  0.43253
year2013      0.13344    0.07981   1.672  0.09455 .
year2014      0.38832    0.07518   5.165 2.40e-07 ***
year2015      0.22031    0.07657   2.877  0.00401 **
year2016     -0.15088    0.20801  -0.725  0.46825
---
Signif. codes:  0 '***' 0.001 '**' 0.01 '*' 0.05 '.' 0.1 ' ' 1

Approximate significance of smooth terms:
                    edf Ref.df Chi.sq p-value
t2(diel,seasonal) 15.55     24  11176  <2e-16 ***
---
Signif. codes:  0 '***' 0.001 '**' 0.01 '*' 0.05 '.' 0.1 ' ' 1

R-sq.(adj) =  0.0796
glmer.ML =  21812  Scale est. = 1         n = 47425
```

```
> summary(gamm.Ar1.SPEYBAY$gam)

Family: binomial
Link function: logit
```

```
Formula:
presence ~ te(diel, seasonal, bs = "cc", k = 6) +  year

Parametric coefficients:
            Estimate Std. Error t value Pr(>|t|)
(Intercept)  -2.99514    0.06353 -47.145  < 2e-16 ***
year2011      0.35454    0.08229   4.308 1.65e-05 ***
year2012     -0.07234    0.10496  -0.689  0.49065
year2013      0.16502    0.08681   1.901  0.05733 .
year2014      0.44923    0.08161   5.505 3.71e-08 ***
year2015      0.26061    0.08335   3.127  0.00177 **
year2016     -0.15373    0.22988  -0.669  0.50367
---
Signif. codes:  0 ‘***’ 0.001 ‘**’ 0.01 ‘*’ 0.05 ‘.’ 0.1 ‘ ’ 1

Approximate significance of smooth terms:
                    edf Ref.df     F p-value
te(diel,seasonal)  22.35     24 40.54  <2e-16 ***
---
Signif. codes:  0 ‘***’ 0.001 ‘**’ 0.01 ‘*’ 0.05 ‘.’ 0.1 ‘ ’ 1

R-sq.(adj) =  0.0471
  Scale est. = 1         n = 47448
```
